# The Longitudinal Assessment of Osteomyelitis Development by Molecular Imaging in a Rabbit Model

**DOI:** 10.1155/2014/424652

**Published:** 2014-09-11

**Authors:** Jim C. E. Odekerken, Geert H. I. M. Walenkamp, Boudewijn T. Brans, Tim J. M. Welting, Jacobus J. C. Arts

**Affiliations:** ^1^Laboratory for Experimental Orthopaedics, Department of Orthopaedic Surgery, CAPHRI School for Public Health and Primary Care, Maastricht University Medical Centre, P.O. Box 5800, 6202 AZ Maastricht, The Netherlands; ^2^Department of Nuclear Medicine, Maastricht University Medical Centre, P.O. Box 5800, 6202 AZ Maastricht, The Netherlands

## Abstract

*Introduction*. Osteomyelitis is a severe orthopaedic complication which is difficult to diagnose and treat. Previous experimental studies mainly focussed on evaluating osteomyelitis in the presence of an implant or used a sclerosing agent to promote infection onset. In contrast, we focused on the longitudinal assessment of a nonimplant related osteomyelitis. *Methods*. An intramedullary tibial infection with *S. aureus* was established in NZW rabbits. Clinical and haematological infection status was evaluated weekly, combined with X-ray radiographs, biweekly injections of calcium binding fluorophores, and postmortem micro-CT. The development of the infection was assessed by micro-PET at consecutive time points using ^18^F-FDG as an infection tracer. *Results*. The intramedullary contamination of the rabbit tibia resulted in an osteomyelitis. Haematological parameters confirmed infection in mainly the first postoperative weeks (CRP at the first 5 postoperative weeks, leucocyte differentiation at the second and sixth postoperative weeks, and ESR on the second postoperative week only), while micro-PET was able to detect the infection from the first post-operative week onward until the end of the study. *Conclusions*. This study shows that osteomyelitis in the rabbit can be induced without use of an implant or sclerosing agent. The sequential follow-up indicates that the diagnostic value of each infection parameter is time point dependant. Furthermore, from all parameters used, the diagnostic value of  ^18^F-FDG micro-PET is the most versatile to assess the presence of an orthopaedic infection in this model.

## 1. Introduction

Currently, posttraumatic and postoperative osteomyelitis remains to be one of the most severe complications after bone trauma or surgery. During the last decades, much research has been conducted into prevention, diagnostics, and treatment modalities for orthopaedic infections. Most research studies focus on treatment or prevention and not on the diagnosis of bone infection. However, novel imaging modalities are made available in the clinical evaluation of osteomyelitic lesions, that is, combined ^18^F-FDG PET and MRI [1].

The preclinical evaluation of any diagnostic tool requires a stable and consistent experimental preclinical model with a broad collection of relevant read-out parameters to yield reliable data with a precise follow-up. In this way, preclinical osteomyelitis models can be highly informative on the development of the disease and the accompanying diagnosis by novel tools like ^18^F-FDG PET [2, 3].

To investigate the development of a nonimplant related osteomyelitis over time and to define a stable preclinical model, we aimed to establish an osteomyelitic lesion in the tibiae of rabbits, without the use of a sclerosing agent, since this destructs the local vascularisation of the bone and reduces the local immune capacity [4, 5].

To investigate the potential of novel diagnostic approaches, we evaluated the sequential use of ^18^F-FDG as an infection specific micro-PET tracer on multiple time points during follow-up.

By combining the results of this study with our previously reported studies [6, 7], we also aimed to identify the most relevant parameters to diagnose naïve osteomyelitis by experimental conditions and describe how these infection parameters may differ in case of the presence or absence of an orthopaedic implant.

## 2. Materials and Methods

### 2.1. Animal Choice, Welfare, and Ethics

Eleven specified pathogen free (SPF) female New Zealand White (NZW) rabbits (Charles River, France), with a weight of 3.5–4 kg (approximately 6 months of age) were used in this study. Animals were allowed to acclimatize for 2 weeks before surgery was performed.

During the study, each animal served as its own control since preoperative measurements had been performed; these functioned as a baseline measurement. The nonoperated contralateral leg was also used as a control for the uncontaminated condition (radiology, micro-PET, and histology). Furthermore, preoperative micro-PET scans were supplemented with a historic control group [7] to reduce the exposure of the animals to ionising radiation in accordance with the ALARA-guideline [8]. Animal housing, feeding, pain treatment, humane endpoints, and sacrifice were performed according to our previously described study [6, 7].

The experimental follow-up scheme is displayed in Table 1.

This study was approved by the Maastricht University Animal Ethics Committee (DEC-UM, protocol 2010-089, Maastricht, the Netherlands).

### 2.2. Animal Surgery and Follow-Up

Animals were anaesthetized according to our previously published study [6]. Subsequently, a hand reamed 4 mm wide defect was drilled into the tibial plateau to open the tibial medullary canal. After reaming, the tibial medullary cavity was flushed with sterile saline to remove bone fragments and haematoma. All animals received intramedullary contamination of 3.8 × 10^5^ CFU* S. aureus* (UAMS-1, ATCC 49230) in 100 *μ*L saline.

The inoculation dose was freshly prepared before surgery from an overnight culture and diluted with sterile saline to an average concentration of 3.8 × 10^5^ CFU per contamination, based on OD600 measurements (Amersham Biosciences, GE Healthcare, USA). The inoculation dose and preparation were based on previous studies [6, 7]. To confirm the inoculum size, the bacterial count of every inoculum was verified by quantitative culture on tellurite glycine agar (Difco, Becton Dickinson, France) before and after surgery.

After contamination, the defect was sealed with bone wax (Syneture, Covidien, USA) and the surrounding tissue was flushed with sterile saline. The patella tendon was apprimated with 4 resorbable sutures and the skin by 6 single intracutaneous inverted sutures (Syneture, Covidien, USA); aluminum-spray (Eurovet Animal Health, the Netherlands) was applied to protect the wound.

The animals were monitored during the 6-week follow-up for the use of their hind legs, the appearance of the wound, and on general signs of infection (redness, swelling, and fever). Body weight and temperature were measured and blood was collected by venipuncture from the jugular vein at the day of surgery and every week thereafter until the end of the experiment. Blood samples were collected weekly and analysed for changes in erythrocyte sedimentation rate (ESR) (Kabe Labortechnik, Germany), leucocyte differentiation (Euregio Laboratory, the Netherlands), and C-reactive protein levels (CRP) (E-15CRP, Immunology Consultants Laboratory, USA).

### 2.3. Radiographic Imaging

Standard X-ray radiographs were collected according to our previously described study [6]. All radiographs were independently scored, by 3 blinded observers, for osteomyelitis mediated bone morphological changes according to our modified scoring system [6].

*Ex vivo* micro-CT imaging of the affected tibiae was performed after 6-week follow-up, directly postmortem. The micro-CT-images were acquired on an X-rad 225 (Precision X-ray, USA) as described previously [6, 7].

### 2.4. ^18^F-FDG Micro-PET

^18^F-FDG micro-PET was conducted to assess the local metabolic glucose uptake in the contaminated tissue, since infection is associated with an increased metabolic glucose turnover. Imaging data was collected by a preoperative scan and three postoperative scans (resp., at one, three, and six weeks after surgery) of each animal.

The entire micro-PET procedure of approximately 2 hours was performed under tiletamine-zolazepam sedation, initiated by a 15 mg/kg intramuscular dose and 3 additional intramuscular injections of 7.5 mg/kg. The time between the first and the subsequent second dose was 20 minutes, all injections following thereafter were given with 45-minute intervals.

The rabbits were fixed in a custom made PVC restrainer, which allowed the rabbit to breath freely without allowing movements of the hind legs [7]. Fifty MBq ^18^F-FDG (GE Healthcare Medical Diagnostics, Eindhoven, the Netherlands) were diluted with sterile saline to a volume of 1 mL and were subsequently injected in the ear vein of the rabbit. Residual activity in the syringe was measured on a CRC-25R dose calibrator (Capintec, USA) to calculate the initially injected ^18^F-FDG dose. A 1-hour incubation period was taken into account to allow uptake of the ^18^F-FDG in the area of interest.

Data were analysed with the ASIPRO VM software package (version 6.7.1.2, Concorde Microsystems, Siemens, Germany). The imaging data were reconstructed by the OSEM2D protocol with an isotropic voxel size of 0.87 mm to align the tibial intramedullary cavity with the coronal, sagittal, and transversal planes to keep the volumes of interest (VOI) of all scans equal. A cylindrical VOI of 10.4 mm in diameter and 25.1 mm long (12 voxels in diameter and 29 voxels long) was used as a contour around the affected bony tissue. An equally sized VOI was placed in the contralateral leg on the equivalent location, to serve as an internal control. Each leg also contained an equally sized VOI in the* vastus lateralis* to serve as a measurement for the soft tissue ^18^F-FDG-uptake. The standardized uptake value (SUV) was calculated from the total activity in the selected VOI, corrected for the weight of the animal and the activity of ^18^F-FDG in the animal at the time of emission scanning. Activity at the time of the scan was corrected for the injected activity and the calibration factor of the micro-PET and dose calibrator.

### 2.5. Bacteriology

After sacrifice, the tibiae were dissected aseptically. Swabs were taken from the knee joint cavity. To assess soft tissue infection, these swabs were evaluated for the presence of* S. aureus* on tellurite glycine agar plates.* S. aureus* growth was identified by the presence of black colonies, due to the coagulase positive character of the species. Other, coagulase negative bacterial species (e.g.,* S. epidermidis*) would appear as white colonies. A 5 mm piece of the distal part of the* tuberositas tibiae* was excised from the tibia with a surgical drill (SM 12, Nouvag, Switzerland). After weight measurement, it was homogenized in 10 mL sterile saline (Ultra-Turrax T25, Ika, Germany) at 6000 rpm. The homogenates were cultured on tellurite glycine agar plates. After 24 hours, culture dishes were quantified for the specific bacterial growth.

### 2.6. Histology

During the experimental follow-up, three different calcium binding fluorophores were administered, by subcutaneous injection, to follow bone apposition and mineralization over time, which are to be detected in histological sections. Injection of Calcein Green (25 mg/kg, Fluka, Sigma Aldrich, Germany) was performed at 2 weeks and Xylenol Orange (30 mg/kg, Fluka, Sigma Aldrich, Germany) at 4 weeks, and Calcein Blue (25 mg/kg, Fluka, Sigma Aldrich, Germany) was injected on the day before sacrifice.

After sacrifice and sampling for bone culture, tibiae were fixated in 4% formaldehyde/PBS for 4 weeks and embedded in polymethylmethacrylate (PMMA) (Technovit 9100, Hereaus-Kulzer, Germany). After polymerization, sections were stained according to Masson-Goldner (Carl Roth, Germany) and Gram (without a safranin counterstain) and subsequently 50 *μ*m sections were obtained using a saw microtome (SP 1600, Leica, Germany). Sections were analysed and digitized by light microscopy (Axioscope A1, AxioVision LE release 4.8.2, Carl Zeiss, Germany). The localization of calcium binding fluorophores in the bony tissue was visualized by fluorescence microscopy (Leica DMRB, Leica IM50 version 1.2 release 19, Leica, Germany) on unstained PMMA sections. Acquired images were merged using Photoshop CS3 (Adobe Systems, USA) to generate overview images.

### 2.7. Statistical Analysis

SPSS 21 (IBM, USA) was used for the statistical analyses. Each animal served as its own preoperative healthy control. Data were checked for normality using the Shapiro-Wilk test. Differences between time points were determined by a Wilcoxon Signed Ranks Test for nonparametric significance. In case of the micro-PET imaging, we randomly selected 3 animals to serve as a healthy preoperative control. The data of these 3 animals were supplemented by a historic control group of 10 animals [7], to reduce overall reduction of animal inconvenience (one-, two-hour episode of anaesthesia in combination with the injection of a 50 MBq ^18^F-FDG). Therefore, the statistical analysis of the micro-PET-data was performed with a combination of the Wilcoxon Signed Ranks Test and the Mann-Whitney *U* Test. The significance level was determined at *P* ≤ 0.05. Graphical representation of the data was performed in GraphPad Prism 5 (GraphPad, USA), and error bars represent standard error of mean.

## 3. Results

### 3.1. Animal Surgery and Follow-Up

Eleven rabbits were intramedullary (right proximal tibia) contaminated with 3.8 × 10^5^ CFU* S. aureus* via a transpatellar tibial plateau administration route. Nine animals completed the 6-week follow-up, which all developed an osteomyelitis in the right proximal tibia after peroperative contamination. One animal did not recuperate from the surgical procedure and another animal was sacrificed 3 weeks after surgery due to complications (humane endpoints were defined as extensive weight loss of >20% and severe soft tissue infection).

### 3.2. *In Vivo* and* Ex Vivo* Data Analysis

**Figure 1:**
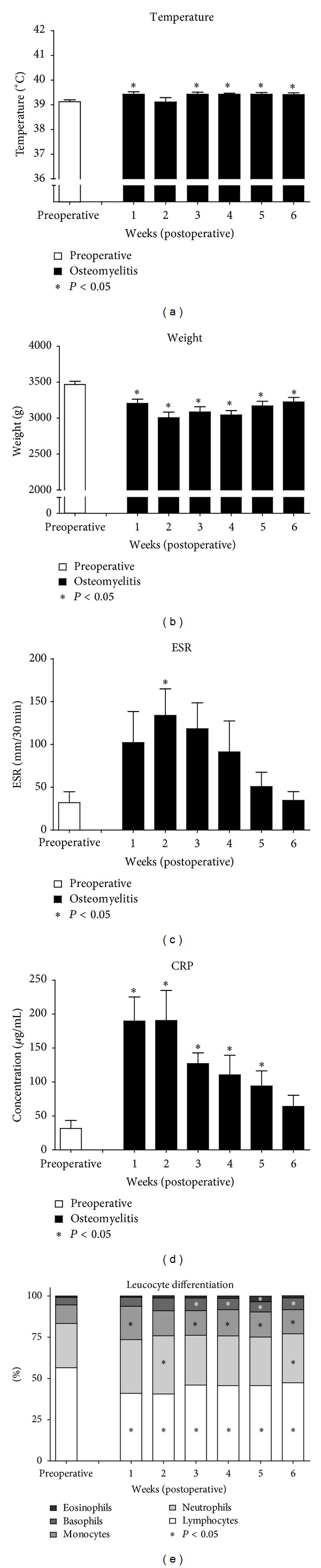
Physiological and haematological parameters. (a) Body temperature during follow-up. (b) Body weight during follow-up. (c) Erythrocyte sedimentation rate. (d) C-reactive protein levels. (e) Leucocyte differentiation. All postoperative values are compared with the preoperative values, in case of significant differences, asterisk indicates *P* ≤ 0.05, and the error bars represent standard error of mean.

After recovery from the surgical procedure, all animals showed limited function of the operated leg with partial weight bearing. Body temperature and weight (clinical indicators for infection) were monitored on a weekly basis. In comparison to the preoperative measurements, a significant increase in body temperature was noted in all but the second postoperative week (Figure 1(a); *P* ≤ 0.049). All animals reduced weight significantly compared to preoperative values and did not regain their preoperative bodyweight values during follow-up (Figure 1(b); *P* ≤ 0.011).

During the six-week follow-up period, the ESR increased during the first weeks after surgery compared to the preoperative rate, with a significant increase only at two weeks after surgery (Figure 1(c); *P* = 0.042).

CRP levels however showed a significant increase in the first 5 weeks after surgery (Figure 1(d); *P* ≤ 0.038).

The weekly assessment of the leucocyte differentiation (Figure 1(e)) indicated that the lymphocytes fraction in the leucocyte differentiation was significantly decreased as compared to the preoperative values on all postoperative time points (*P* ≤ 0.017). The neutrophilic granulocyte fraction was only significantly different on the second and sixth postoperative week (*P* ≤ 0.050). The monocyte fraction was significantly increased in all but the second postoperative week (*P* ≤ 0.033). The basophilic granulocyte fraction was significantly increased from the third postoperative week onward (*P* ≤ 0.038). The eosinophilic granulocyte fraction was only significantly different at the fifth postoperative week (*P* = 0.042).

**Figure 2:**
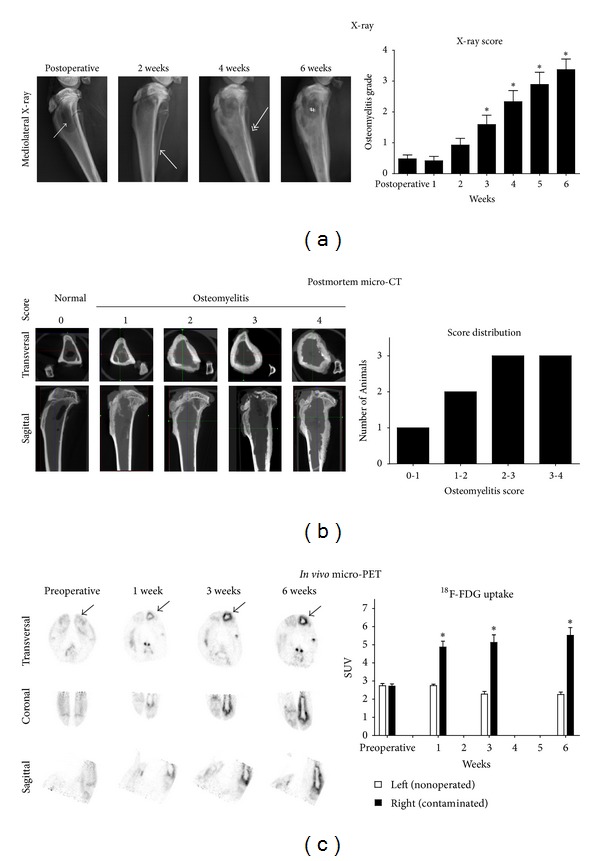
Imaging data. (a) Sequential X-ray radiographs during follow-up. The surgical procedure can result in radiologic symptoms for osteolysis (small arrow head). Two weeks after surgery, periosteal elevation can be detected (solid arrow head). After four weeks, cortical thickening (double headed arrow) and osteolysis can be detected (asterisk). At six weeks, the osteolysis is affecting the entire proximal part of the tibia (hash sign). Graph depicts the increase in radiological score during the developmental stage of an osteomyelitis. Radiological scores during follow-up were compared to the preoperative radiological score, asterisk indicates *P* ≤ 0.05, and error bars indicate standard error of mean. (b) Representative postmortem micro-CT images taken after the six-week follow-up. (c) Reconstructed ^18^F-FDG micro-PET data shows an increased tracer uptake in the operated area during the experimental follow-up (arrow). Asterisk indicates *P* ≤ 0.05 and error bars indicate standard error of mean.

The bone morphological changes initiated by the peroperative contamination of the tibia were detectable on X-ray radiographs from the 3rd postoperative week onwards (Figure 2(a)), when scored according to our previously described osteomyelitis scoring system [6], focusing on bone morphological changes like osteolysis, periosteal elevation, and cortical thickening. The micro-CT data (Figure 2(b)) allowed post-mortem assessment of the bone morphological changes as a result of the established osteomyelitis, confirming the X-ray data. The normal condition shows a clearly defined cortex without apparent signs of osteolysis, and the osteomyelitis conditions shows cortical thickening, loss of cortex integrity, and signs of moderate to severe osteolysis. The graph depicts the distribution of the radiological score in the experimental group.

Quantification of the micro-PET tracer uptake indicated that there was no statistical difference in tracer uptake in the proximal part of the tibia between the experimental preoperative group and the historic control group [7] (left leg *P* = 0.371; right leg *P* = 0.112).

There was no statistical difference in tracer uptake between the left and right leg of the combined preoperative control group (including the historic control group) (*P* = 0.753).

The ^18^F-FDG uptake in the operated right leg was significantly increased at all postoperative time points compared to the uptake in the right tibia of the combined preoperative control group (Figure 2(c), preoperative black bar compared to postoperative black bars; *P* ≤ 0.001).

All postoperative time points showed an increased ^18^F-FDG uptake, indicated by the standardized uptake value (SUV) in the operated and contaminated right leg when compared to the uncontaminated left leg (Figure 2(c), white bars compared to black bars; *P* ≤ 0.012).

**Figure 3:**
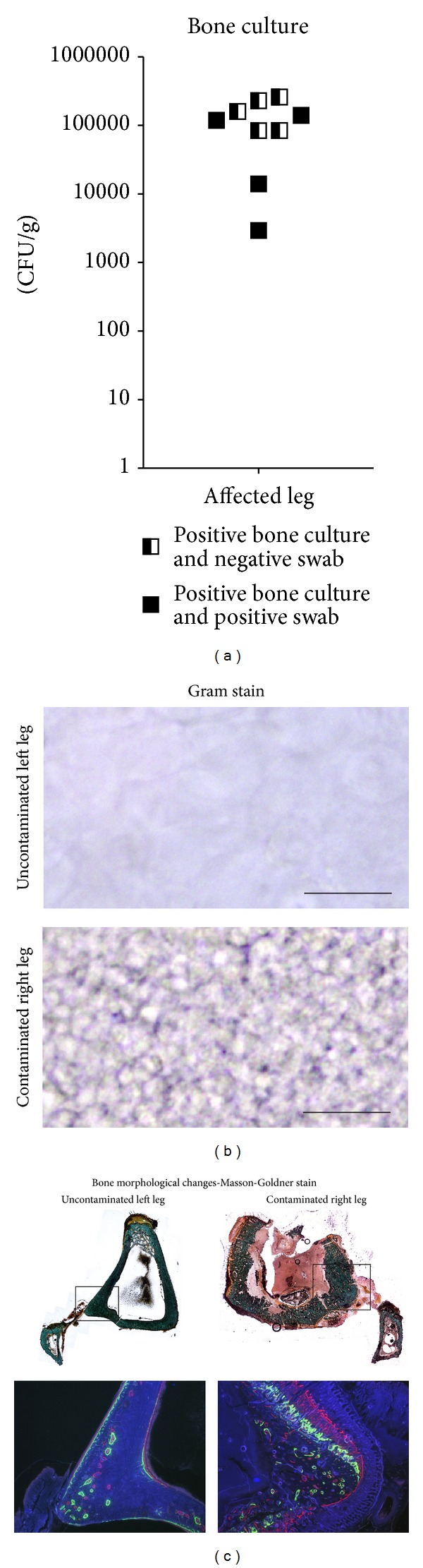
Bacterial culture and histology. (a) Postmortem bacterial culture of the excised part of the tibial tuberosity of the osteomyelitic tibia, indicating bacterial presence in all tibiae. (b) Histological confirmation of bacterial presence, by Gram stain, in the contaminated right leg only. (c) Bone morphological changes between left and right tibia. Tibial tuberosity is missing in the section of the right leg since this was used for bacterial culture. Fluorescence microscopy (insert) indicated normal bone remodelling in the left tibia, while it indicates cortex remodelling during the experimental follow-up in the right tibia (green at two weeks, red at four weeks, and blue at six weeks after surgery).

Indicative for an active osteomyelitis, bacterial cultures of swabs taken from the knee joint cavities tested positive for* S. aureus* in 4 out of 9 cases, while bone tissue homogenate cultures tested positive for* S. aureus* in all cases (Figure 3(a)).

Histological staining of PMMA sections of the tibiae indicated the presence of Gram-positive cocci in the intramedullary cavity of the contaminated tibiae only (Figure 3(b)). Masson-Goldner staining indicated a clearly defined well-structured cortex, without signs of periosteal elevation or cortical thickening in uncontaminated left tibiae, while the contaminated right tibiae showed cortical thickening and loss of cortical integrity, indicating osteomyelitis (Figure 3(c)).

The state of bone remodelling was assessed by calcium binding fluorophores. In normal aseptic bone remodelling closely matched fluorescent signals (calcium deposition) are to be expected, whereas diseased bone remodelling would show calcium deposition in an outward direction in clearly defined layers (green: 2 weeks after operation, red: 4 weeks after operation; blue: the day before sacrifice). This allowed a clear discrimination between the normal left (unoperated and uncontaminated) and the right (operated and infected) tibia of all animals. The calcium binding fluorophores indicated undisputable signs of periosteal elevation and cortical thickening, in the infected tibiae (Figure 3(c)); these hallmarks for infection are confirmed by our previously published studies concerning orthopaedic implant infections.

## 4. Discussion

Osteomyelitis remains to be a major complication after an orthopaedic intervention or after bone trauma [9, 10]. The herein described study was conducted to provide insight into osteomyelitic development including the accompanying bone remodelling and the use of novel diagnostic approaches in the absence of an orthopaedic implant and a sclerosing agent to support infection development. Combining the results of this study with our previously published studies on orthopaedic implant infection [6] and the use of ^18^F-FDG to detect implant infection by PET-imaging [7], we can provide an improved perspective on how the used infection parameters differ from each other in case of either implant or non-implant related orthopaedic infections.

Animal models for experimental (implant-related) osteomyelitis, like ours, are being used to evaluate the application resorbable biomaterials and antimicrobial coatings and their antimicrobial properties [11–14]. For this reason, no sclerosing agent like sodium morrhuate was used, since it poses a threat to resorbable biomaterials, due to the denaturing capacity of sodium morrhuate [15–18]. Furthermore, sodium morrhuate creates an abnormal bone anatomy by destructing the local vascularization [5, 19].

Common haematological parameters like ESR, CRP, and leucocyte differentiation are potent parameters for infection detection. However—there are some practical difficulties—the ESR is difficult to determine due to the used capillary detection system in our experimental setup, resulting in large deviations at the moment of readout. This issue makes this method of determination less accurate. The determination of the CRP concentration on the other hand is performed by ELISA, which is a very sensitive method, resulting in a more accurate readout. However, both ESR and CRP show the same trend, which is increased levels in the first weeks after surgery, after which the levels slowly decrease.

The leucocyte differentiation however shows a specific increase or decrease of a specific fraction of the leucocyte pool. For example, acute infection is generally related to a decrease in the lymphocyte fraction and a related increase in favour of the neutrophil and monocyte fraction. When we combine these haematological findings with the fact that the infection remains active (even when the ESR and CRP are decreasing but the shift in leucocyte differentiation remains present) our data suggests that the infection is stabilizing. This finding was confirmed by our previously performed study on orthopaedic implant infections.

X-ray radiographs allow detection of bone infection due to morphological changes like infection-initiated mineralization and osteolysis. Radiography is often used on single time points. The use of multiple imaging moments during follow-up allows monitoring of the bone morphological changes in the affected region. By detecting periosteal elevation, focal loss of cortex integrity, and osteolysis, an osteomyelitic lesion can be detected in time during radiological follow-up and thus allows monitoring of infection progression, whereas the presence of an implant may disturb radiologic detection of infection hallmarks due to implant related scatter of the X-rays; nonetheless, the influence (on radiologic imaging) of the implant will remain equal during follow-up which will not hamper the sequential evaluation of bone mineralization and morphology over time.

^18^F-FDG has been described as a clinical PET tracer for metabolic active processes (e.g., tumour growth, brain activity) [20, 21]. Together with previously published data of others, our data indicates the diagnostic potential of ^18^F-FDG as a micro-PET tracer for the detection of osteomyelitis in relation to other relevant infection parameters, especially since ^18^F-FDG micro-PET indicates abnormal metabolically active areas in the body [2, 7, 22]. Furthermore, our data indicates that ^18^F-FDG micro-PET allows differentiation between contaminated and uncontaminated bony tissue within one week after surgery. Due to the ability of ^18^F-FDG micro-PET to distinguish between infected and uninfected tissue, this could be a powerful tool to assess novel infection interventions (coatings, antibiotic treatments). However, previously published studies have shown that the presence of an implant or an osteotomy/fracture can hamper early detection by PET due to implant-related scatter of the excited photons on the metallic implant surface or by tracer uptake due to a local sterile inflammation (fracture) [7, 22]. This indicates that the use of ^18^F-FDG is a very sensitive method to detect increased metabolic activity in the bony tissue; however, the specificity is dedicated not only towards infection but also towards sterile inflammation due to tissue damage (by a surgical intervention or fracture) [7, 22].

Bacterial culture is considered the golden standard for the detection of active bone infections [23–25]. The additional histological analysis for bacterial presence, by Gram-staining, of the PMMA sections of the tibiae indicates the presence of bacteria in the affected tissue, while standard histological stainings indicate the bone morphological changes initiated by the bacterial infection.

Calcium binding fluorophores have been used in the past to follow bone remodelling and the mineralization of teeth [26–30]. In contrast to these previous studies, our data shows that these calcium binding fluorophores can also be of considerable value to monitor and quantify osteomyelitis related bone remodelling, specifically the mineralization of the periosteal elevation [6]. Furthermore, when these data are combined with the haematological data, they show that the infection-mediated bone mineralization remains progressing even after the ESR and CRP levels are decreasing, strengthening the indication for an acute stabilizing (potentially chronic) infection. Furthermore, when combined with our previously published data [6, 7], these data indicate that these changes in bone remodelling are infection dependent and not associated with the presence nor absence of an implant.

Our collected data provides novel insight into osteomyelitis development and suggestions of parameter usage in both preclinical and clinical perspectives. In the preclinical setting, the body weight and temperature provide general information about the condition of the animal and should be regarded as such. The use of weekly assessment of the CRP levels and leucocyte differentiation, in combination with weekly X-ray radiographs, histology, and bacterial culture, is recommended. Although ^18^F-FDG microPET, post-mortem microCT and calcium bindingfluorophores provide optional information on the infection and its related bone mineralization, they are not absolutely necessary to determine if an antimicrobial coating or biomaterial is effective to prevent an osteomyelitis. While a combined follow-up with X-ray radiographs, histology, bacterial culture and haematological analysis will provide sufficient information to determine antimicrobial efficacy. However, when interested in prophylactic approaches, the determination of delayed onset of the actual infection, or infection mediated bone remodelling and osteolysis, ^18^F-FDG micro-PET, and postmortem micro-CT, and the use of calcium binding fluorophores provide more in depth information about timing, metabolic activity, and bone mineralization.

When translated to the clinical situation, however, the situation is different, and CRP and leucocyte differentiation will still be useful and so will X-ray radiographs, but calcium binding fluorophores are not an option. Based on our data, ^18^F-FDG PET (combined with CT/MRI in one clinical system) could considerably contribute to the early detection of an orthopaedic infection allowing early infection intervention and treatment in the clinical situation.

## 5. Conclusion

Our study describes the detection of different bone infection parameters and their correlations in an experimental osteomyelitis animal model (independent of the presence of an implant) and provides information on which parameters would be the most optimal infection parameters to be of use in the preclinical and potentially the clinical setting. Furthermore, our study showed that ^18^F-FDG PET is a potent diagnostic tool for the early detection of orthopaedic infections, which can be of great value when applied in the clinical situation.

## Figures and Tables

**Table 1:** Experimental parameters and follow-up.

	Quantification inoculum	Weight	Temperature	ESR	CRP	Leucocyte differentiation	X-ray	Micro-CT	^ 18^F-FDG micro-PET	Calcium binding fluorophores	Bone tissue culture	Histology
Preoperative		X	X						X			
Day of surgery	X	X	X	X	X	X	X					
Week 1		X	X	X	X	X	X		X			
Week 2		X	X	X	X	X	X			X		
Week 3		X	X	X	X	X	X		X			
Week 4		X	X	X	X	X	X			X		
Week 5		X	X	X	X	X	X					
Week 6		X	X	X	X	X	X		X	X		
Postmortem								X			X	X
